# Over-Worked Matrons

**Published:** 1908-02-15

**Authors:** 


### Nursing Administration.

### OVER-WORKED MATRONS.

It is a curious phenomenon that in proportion as the duties appertaining to hospital matrons tend to become more and more difficult to discharge, the posts are the more eagerly competed for. The position has its dangers. Excessive competition tends undoubtedly to lower the conditions of work in any employment. Competition in this direction shows a tendency to keep the rates of salary very low, and it may also have its effect in making these posts unduly arduous. We pointed out a short time ago that the proper course for an overworked matron to pursue was to resign, having first clearly stated to her committee the difficulties under which she laboured; but a correspondent demurs to this on the ground that for every matron who resigns there are hosts of applicants eager to step into her shoes and attempt the impossible. We are fully satisfied that no inducement of salary, no anxiety about future prospects should compel a woman who is aware that her health is being ruined by her work to continue in office. Even on economic grounds she is in a far better position among the unemployed, in full vigour, ready for a new post, than lingering out weary months in a rest-cure home, permanently enfeebled, as so many workers become, by long hours and incessant worry. Moreover, the over-worked matron has a duty to the institution. An ignorant committee may ignore the representations of one matron, but no committee will be found to disregard the protest, albeit a silent one, enforced by the resignation of two or three valuable officers successively. To leave the security of a good post and take a leap in the dark certainly demands a high degree of courage. It is for this reason that so often the over-worked matron goes drifting on, suffering more than any one can ever know, until nature takes the matter into her own wise hands and calls out unmistakably for an end to the situation.

The matter lies in reality in a nutshell. In ninety-nine cases out of a hundred the matron is overworked for want of an assistant. We have examined the actual position of affairs in training schools containing over a hundred beds, in that useful volume, "How to Become a Nurse." In London there are twelve hospitals with an assistant matron or the equivalent, a home sister, and five without. Among Poor-law infirmaries in London, twenty have an assistant matron and two have not. Among provincial hospitals thirty-two have an assistant matron or home sister, and twenty-eight have none. Among provincial Poor-law infirmaries with training schools, fifteen have an assistant matron and three have none. Among the larger Scotch hospitals seven have an assistant matron and five have none. In Ireland six have an assistant matron and four have none. These figures are altering every year. We know of no instance in which the services of an assistant matron have been dispensed with when they have once been enjoyed. But year by year the laggards are coming into line, and one institution after another grows to recognise the importance of setting at the matron's disposition the entire time of a senior sister, to assist her in supervision, in housekeeping, and in office work, and to relieve her in necessary off-duty hours. Under whatever name this extra sister is known she is the only effectual instrument through whom the matron can obtain assistance worthy of the name. The day's work of the matron in hospitals with training schools attached is indeed, if it be studied critically with understanding of its conflicting claims, entirely beyond the ability of one pair of hands to discharge as it ought to be discharged. It is unfortunate that so few lay members of hospital committees have any opportunity of getting to realise this fact. They are often curiously ignorant of the routine of the institution which they help to govern. A small volume has recently been published by the Scientific Press called "The Matron: Her Duties and Responsibilities." We could wish it were circulated freely among committees and boards of management that governors might come to a clear understanding not only of the intricate matters which fall to the matron's share, but also of the extent to which the hospital depends for efficiency and economy on the matron's facilities for carrying out inspection thoroughly. Now it is quite certain that if the matron is overworked it is the inspection which must perforce be neglected. When a woman is torn asunder by the knowledge that several duties claim her at once the duty which can most safely be left for the moment is the duty of examining into the manner in which someone else is doing her work. That can be deferred. Accordingly when there is no assistant matron much is often left uninspected which cries out for the searching eye of the matron. Dinner must be ordered, books must be made up, letters must be written, people must be interviewed; the list of the "musts" could be indefinitely expanded. But nothing dreadful will happen if the matron does not inspect the larders at her usual hour, or if she fails to pay her usual evening visit to the wards, or leaves the linen unchecked one week. And so the system gets a little slack, careless sisters reckon on her not having time to see this or that, and the matron is burdened day and night with a sense of duty unperformed. This is the worry that kills.

It is not extra nurses which are needed, far less extra probationers, when a general condition of overwork pervades the institution. Nearly always the worry is occasioned by the necessity for disorganising one ward after another to set a sister free to do things for matron, or to superintend the out-patient work, or to attend to some emergency. In such cases one extra sister is worth half a dozen subordinates. And we venture to maintain that the modern institution with its high standard of efficiency towards which all must strain, cannot be properly supervised unless the matron have the use of at least one sister to act solely as her assistant.

